# Long-Term Cancer-Free Survival after Infusion of Autologous Cord Blood Cells for Rejection-Unrelated Cord Blood Graft After Myeloablative Conditioning for t-MDS: A Case Report

**DOI:** 10.1097/MD.0000000000000438

**Published:** 2015-02-06

**Authors:** Wei-Ting Chen, Li-Hua Fang, Rong-Long Chen

**Affiliations:** From the Departments of Internal Medicine (W-TC); Pharmacy (L-HF); and Pediatric Hematology and Oncology (R-LC), Koo Foundation Sun Yat-Sen Cancer Center, Taipei, Taiwan.

## Abstract

Autologous cord blood transplantations are rarely applied in patients with hematologic as well as metastatic solid cancers since contamination of malignant clones is a concern.

We report a case of therapy-related myelodysplasia after metastatic neuroblastoma who suffered from graft rejection and life-threatening infections after a myeloablative unrelated cord blood transplantation.

The patient experiences long-term survival, free from leukemia/neuroblastoma after infusion of autologous cord blood cells.

It suggests that autologous cord blood transplantation after high dose therapy might be a curative strategy for certain hematologic or metastatic solid cancers.

## INTRODUCTION

Therapy-related myelodysplastic syndrome/acute myeloid leukemia (t-MDS/AML) after neuroblastoma typically are drug resistant with poor outcome whereas allogeneic stem cell transplantation can offer long-term event-free survival.^1^ Unrelated cord blood transplantation (CBT) has successfully been used for treatment of leukemia in young children; however, it still carries a substantial risk of potentially fatal graft failure.^2^ Herein, we report a case of t-MDS postneuroblastoma, who suffered from graft failure with unstable clinical condition after a human leukocyte antigen (HLA) 4/6-matched unrelated CBT. As only low dosage of autologous cord blood cells was available, she emergently received the cells mainly through direct intramedullary injection, which resulted in complete hematopoietic recovery accompanied with remission of her leukemia.

## CASE REPORT

A 4 year and 8 months old girl was diagnosed with a stage-4 neuroblastoma of right adrenal primary with bone marrow and multiple bone metastases in October 2006. She received 6 cycles of intensive induction chemotherapy according to the Memorial Sloan-Kettering Cancer Center N7 protocol.^3^ Subtotal resections of the right adrenal tumor and metastatic lymph nodes were performed in June 2007 with massive postoperative retroperitoneal hematoma resulting in obstructive jaundice and mechanical ileus that required total parenteral nutrition. Therefore, 2 more cycles of topotecan and cyclophosphamide were given. Because Children's Oncology Group phase III data from the A3973 trial showed no advantage in event-free or overall survival with a purged versus an unpurged peripheral blood stem cell (PBSC) product,^4^ she then was conditioned by high-dose therapy with carboplatin (1700 mg/m^2^), etoposide (1352 mg/m^2^), and melphalan (210 mg/m^2^) followed by unpurged autologous PBSC infusion with a dosage of CD34^+^ cells being 3.9 × 10^6^/kg on October 4, 2007. Neutrophil engraftment was documented on day +12 and transfusion independency was achieved after day +9. Local irradiation with a dosage of 2160 cGy over defined areas of the abdomen and left skull bones was also administered and completed on November 21, 2007.

She subsequently received 6 cycles of oral 13-cis retinoic acid followed by 5 monthly intravenous Zometa (Novartis, Schaffhauserstrasse, Switzerland) (2 mg/m^2^) plus daily oral thalidomide (100 mg) until November 2008. However, the blood counts [white blood cell (WBC) 3200–4500/μL, neutrophils 800–2800/μL, hemoglobin (Hb) 9–10.4 g/dL, and platelets (plts) 41,000–67,000/μL] were suboptimal during the period, though no transfusions were required. The pancytopenia worsened further in December 2008. World Health Organization refractory anemia with excess blasts, type 1 was diagnosed after bone marrow studies on December 24, 2008 showed the presence of 8% blasts with characteristic dysplastic changes of myeloid, erythroid, and megakaryocytic lineages. The cytogenetic analysis of bone marrow cells showed clonal expansion of cells with a 46 XX, del(7)(q22), der(9)t(9;?)(q34;?) karyotype in 6 of the 20 metaphases.

A decision was made to pursue unrelated CBT for salvage. The conditioning treatment consisted of fludarabine (160 mg/m^2^) and intravenous busulfan (18 mg/kg) divided in 4 daily doses as well as 5 mg/kg thymoglobulin divided in 3 daily doses. For prophylaxis against graft versus host disease, she received tacrolimus (starting day −3) and methylprednisolone (starting day +5). On 2 March 2009, she received a single unit of unrelated cord blood cells containing 5.6 × 10^7^ total nucleated cells (TNC)/kg and 2.5 × 10^5^ CD34^+^ cells/kg. The patient and donor were HLA-4/6 matched and ABO-nonidentical (A to B). The postinfusion course was complicated by an episode of *Streptococcus viridans* sepsis. Although transient-mixed chimerism could be documented between day +7 and day +14 with WBC rising to 600/μL on day +8, complete recipient chimerism was found with a persistently low WBC count of 100/μL after day +14. Perianal erythema was noted on day +28. Fever and rapid clinical deterioration were noted the next day, including renal dysfunction and shock with low oxygen saturation that required intensive monitoring, inotropic agents, and oxygen supplementations from day +29 to day +32. The vital signs stabilized after adjustment of parenteral antibiotics by intensive measures; however, the painful erythematous skin lesions extended gradually over the buttock and perineum.

Although she had autologous cryopreserved cord blood cells (Bionet Corp, Taipei, Taiwan) available, the dosage was low, with TNC of only 22.7 × 10^7^. Because of the graft failure with persistent complete recipient chimerism and because of her critical condition, direct intramedullary transplantation of a low-dosage (TNC being 0.86 × 10^7^ per kg) autologous cryopreserved cord blood was used for salvage on April 9, 2009 (day 0, corresponding to day +38 after unrelated CBT). Modified from a published protocol,^5^ the autologous cord blood unit was thawed in a 37 °C water bath, washed, and suspended in 20 mL saline solution containing dextran and human albumin, and aliquoted into five 4-mL syringes. With intravenous anesthesia using propofol, two 4-mL aliquots each were injected into the bone marrow spaces of the right and left posterior iliac crests, respectively. The remaining 4-mL aliquot was infused intravenously through Port-A. After the transplant, the clinical condition stabilized gradually, although the hematologic recovery was slow. Stable autologous neutrophil engraftment was finally documented on day +65 after autologous cord blood transplant. No transfusions were required after day +99. The platelet counts reached >20,000 and 50,000/μL on day +126 and +141, respectively. The most recent blood count was normal: WBC >4500/μL, neutrophils >2500/μL, Hb 10.7 to 12.1 g/dL, and plts >197.000/μL. Follow-up bone marrow cytogenetic studies all showed 100% 46, XX karyotype. Now, she is alive and free of neuroblastoma/leukemia >5 years after the autologous cord blood transplant.

## DISCUSSION

The conditioning regimen used in our patient does not reliably facilitate engraftment of dual umbilical cord blood grafts in adult recipients.^6^ Although rescue with a second cord blood unit has been successful in the treatment of graft failure, the procedure requires additional conditioning therapy that might place patients at risk of organ dysfunctions and infections.^7^ Successful autologous stem cell transplantation using cells harvested prior to the development of the secondary malignancy has been reported in a small number of patients with t-MDS/AML.^8^ Salvage autologous stem cell transplantation for engraftment failure after allogeneic stem cell transplantation for a case of t-MDS/AML with primary rhabdomyosarcoma has been reported; unfortunately, the patient died of acute renal failure and failure to engraft.^9^ As direct intramedullary CBT has been shown to overcome graft failure even with low numbers of cord-blood cells are transplanted,^5^ we decided to use the available low dosage autologous cord blood cells for our patient in order to boost blood cell recovery. The subsequently seen blood cell recovery was slow but eventually complete. It is unclear how much of the recovery can be attributed to the autologous cord blood cell infusion and how much was due to endogenous marrow cells that had survived the conditioning regimen. What was noteworthy, however, was that the patient's malignant cell clone, which represented approximately one-third of the dividing cells, has remained undetectable for now up to 5 years after the rescue with autologous cord blood cells. Moreover, the patient's neuroblastoma has remained in complete remission.
